# Editorial: Further Understanding of Serotonin 7 Receptors' Neuro-psycho-pharmacology

**DOI:** 10.3389/fnbeh.2015.00307

**Published:** 2015-11-13

**Authors:** Carla Perrone-Capano, Walter Adriani

**Affiliations:** ^1^Pharmacy, University of Naples Federico IINaples, Italy; ^2^Institute of Genetics and Biophysics “A. Buzzati Traverso”, National Research CouncilNaples, Italy; ^3^Department of Cell Biology and Neurosciences, Istituto Superiore di SanitàRome, Italy

**Keywords:** 5-HT7 receptor, signal transduction pathways, central nervous system, neurodevelopmental disorders, neuroplasticity

This volume assembles a number of original research articles and review papers focused on the serotonin receptor 7 (5-HT7R), the most recently described member of the serotonin receptor family.

The idea of making this volume came out at the end of a Workshop held in Rome on September 2013. The aim of the Workshop was to bring together researchers working on the emerging neurobiological roles played by 5-HT7R. Since then, novel knowledge on this receptor continues to be revealed (Gellynck et al., 2013; Nikiforuk, 2015; Santello and Nevian, 2015).

Molecular modeling and site-directed mutagenesis experiments led to the identification of essential residues, which are important for receptor-ligand binding and G-protein activation in the human 5-HT7R (Impellizzeri et al., 2015). This seven-transmembrane domain receptor is positively linked to adenylate cyclase through the stimulatory Gαs protein and is also coupled to Gα12 protein to activate small GTPases of the Rho family. Guseva et al. (2014) provide an overview of the molecular mechanisms responsible for the 5-HT7R -mediated signaling. They discuss in detail the involvement of 5-HT7Rs in regulating different cellular and subcellular processes, as well as the pharmacological properties of these receptors.

Speranza et al. (2015), using neuronal primary cultures dissociated from various areas of the CNS, demonstrate that stimulation of 5-HT7R enhances neurite outgrowth through several signal transduction pathways, such as mTOR, the Rho GTPase Cdc42, Cdk5, and ERK: all these do converge to modulate cytoskeleton reorganization.

*In vitro* studies were also exploited by Samarajeewa et al. (2014) to show that 5-HT7R activation promotes an increase in TrkB receptor expression and phosphorylation, involving pathways downstream of both Gαs and Gα12 protein. Since TrkB is one of the receptors for BDNF, these results raise the interesting possibility that the BDNF signaling might be modulated by 5-HT7R activation also *in vivo*.

The role of 5-HT7R in living animals has been addressed by various papers in this volume. Developmental-sensitization studies have been performed in rats subchronically treated with a brain-penetrant and selective 5-HT7R agonist (LP-211, Hedlund et al., 2010): rats were exposed during adolescence, and acutely probed with the same drug at adulthood (Altabella et al., 2014). By using pharmacological MRI and immunohistochemical analyses, the authors show that LP-211 administration during adolescence induces physiological changes in the septal 5-HT7R expression and astrocyte response that persist also into adulthood.

The importance of 5-HT7R in modulating the circadian rhythm is highlighted by experiments showing that selective pharmacological blockade of this receptor attenuates both photic and non-photic phase shifts of circadian wheel running activity in mice (Shelton et al., 2015). These results may indicate a potential role played by this receptor in the mutual interaction between circadian rhythm desynchronization and depression.

Although it is well-recognized that modulation of 5-HT7R affects learning and memory (Freret et al., 2014), recent investigation on this topic is somehow contradictory, as reviewed by Meneses (2014). Several aspects, such as the effects of agonists or antagonists, as well as the role of 5-HT1A/5-HT7 dimerization, need to be further investigated. Although it seems a reliable finding that the stimulation of 5-HT7R leads to increased levels of cAMP and to improved memory, further work is required for better understanding the functional complexity of the 5-HT7R in memory formation and amnesic conditions.

The involvement of 5-HT7R on complex cognitive functions is reviewed also by Beaudet et al. (2015), with emphasis on hippocampus-dependent memory processing. They show that 5-HT7R plays a major role in allocentric (externally-centered), but not egocentric (body-centered), spatial navigation. These findings are particularly interesting in a translational perspective, since allocentric navigation declines with age and in patients with Alzheimer's disease. Understanding the role of 5-HT7R in spatial memory may allow the identification of novel targets for pharmacological treatment of age-related cognitive decline.

Numerous recent data indicate that 5-HT7R modulates the neuronal morphology, excitability, and plasticity, hence contributing to the establishment of brain connectivity during embryonic and early postnatal life. The results reviewed by Volpicelli et al. (2014) strongly suggest that the 5-HT7R-mediated remodeling of neuronal morphology and circuitry may still occur in the mature brain. As a consequence, this receptor may represent a promising target for innovative therapeutical strategies in animal models of several neurodevelopmental and neuropsychiatric disorders associated with abnormal CNS connectivity.

In this direction, Costa et al. (2015) show that activation of 5-HT7R by new selective agonists reverses metabotropic glutamate receptor-mediated long-term depression (mGluR-LTD) in wild-type and in Fmr1 KO mice, a mouse model of Fragile X Syndrome, with abnormal enhancement of mGluR-LTD. Thus, the activation of 5-HT7R appears to restore synaptic plasticity, suggesting that agonists of this receptor might be envisaged as a novel therapeutic strategy for Fragile X Syndrome. Another neurodevelopmental disease that might benefit from drugs targeting the 5-HT7R is Rett Syndrome (RTT), a disorder in which severe symptoms affect cognitive, sensory, emotional, motor and autonomic functions. Here, de Filippis et al. (2015) show that repeated systemic treatment with the 5-HT7R agonist LP-211, rescues RTT-related deficits in motor coordination, spatial reference memory, and hippocampal synaptic plasticity, as observed in a female mice model of RTT.

Finally, recent key findings indicate that this receptor has also pathophysiological relevance and therapeutic potential in intestinal inflammatory conditions, such as various gut disorders and enteric infections. In the gut, the 5-HT7R is expressed by smooth muscle cells, enteric neurons, enterocytes, and immune cells. As reviewed by Kim and Khan (2014), its stimulation can influence muscle tone and enteric neuron excitation and can promote inflammation by activation of dendritic cells.

Altogether the findings presented in this volume witness the multidisciplinary efforts that are currently in progress, to provide new insights into the morphological, molecular, and functional role of the 5-HT7R in the brain and in the gastrointestinal tract. Hopefully, this book will set the ground for the development of new drugs targeting this receptor as a novel therapeutic strategy in CNS and gut disorders.

## Author contributions

CPC wrote and supervised the Editorial. WA supervised the Editorial.

### Conflict of interest statement

The authors declare that the research was conducted in the absence of any commercial or financial relationships that could be construed as a potential conflict of interest.
